# A109 PATIENTS’ DESCRIPTIONS OF THEIR LAST BOWEL MOVEMENT BEFORE COLONOSCOPY CORRELATES STRONGLY WITH THEIR BOSTON BOWEL PREPARATION SCALE (BBPS) SCORE WHEN ASSESSED USING A STANDARDIZED METHOD

**DOI:** 10.1093/jcag/gwab049.108

**Published:** 2022-02-21

**Authors:** O Akman, J C Gregor, H Singh

**Affiliations:** 1 ^1.^ Gastroenterology, University of Manitoba, Winnipeg, MB, Canada; 2 ^2.^ London Health Sciences Centre, London, ON, Canada; 3 ^3.^ University of Manitoba, Winnipeg, MB, Canada

## Abstract

**Background:**

Predicting bowel prep quality prior to colonoscopy may help improve colonoscopy prep quality by instituting adjunct measures before colonoscopy. This will in turn reduce repeat procedures, complications, and costs.

**Aims:**

We determined the utility of a standardized method to obtain information from patients on the characteristics of their last bowel movement before colonoscopy by correlating the obtained information to BBPS. We compared this approach to informal/usual descriptions attained by nurses.

**Methods:**

This is a cross-sectional study with data collected at two tertiary care hospitals. On the day of their colonoscopy, outpatients were asked by an assistant to describe their last bowel movement using a standardized method. The same patients had previously been asked by endoscopy nurses to describe their last bowel movement; the assistant was blind to previous descriptions. Descriptions were assigned a score (Table 1).

Following colonoscopy, total and segmental BBPS scores were recorded by the gastroenterologist performing the procedure; the gastroenterologist was blind to the descriptions.

Bivariate Pearson’s Correlation was used to separately assess the correlation between descriptions and BBPS. Cohen’s Kappa was used to assess agreement between the two descriptions.

**Results:**

121 patients (ages 17–86; 55% Female) with 11% BBPS< 6 were included.

For descriptions attained by the assistant, there was a strong correlation with total BBPS score (r = 0.738; p < 0.001); correlation to segmental BBPS was moderate-to-strong (r=0.70, 0.63 and 0.67 right, transverse and left colon respectively).

For descriptions attained by endoscopy nurses, there was a moderate correlation with total BBPS score (r = 0.525; p < 0.001); correlation to segmental BBPS was weak-to-moderate (r=0.52, 0.49 and 0.44 right, transverse and left colon respectively).

There was weak agreement (K = 0.525) between description attained by assistant and nurses.

**Conclusions:**

When asked using a standardized method, a patient’s description of their last bowel movement correlates strongly with their total and segmental BBPS score; the correlation is weaker for non-standardized descriptions attained by endoscopy nurses. A standardized questionnaire, including assessing consistency, is valuable in assessing the bowel movement before colonoscopy.

**Funding Agencies:**

None

**Table:** Scores Assigned to Descriptions

**Description to Assistant**	**Score Assigned**
Fully Solid	0
Liquid with Brown Pieces	1
Brown Liquid	2
Clear / Yellow Fluid	3
**Description to Nurse**	**Score Assigned**
Brown	0
Yellow	2
Clear	3

